# The pharmacokinetics and pharmacodynamics of fentanyl administered via transdermal patch in horses

**DOI:** 10.3389/fpain.2024.1373759

**Published:** 2024-03-20

**Authors:** Rachel A. Reed, Londa J. Berghaus, Rose M. Reynolds, Brittany T. Holmes, Anna M. Krikorian, Daniel M. Sakai, Yushun Ishikawa, Heather K. Knych

**Affiliations:** ^1^ Department of Large Animal Medicine, University of Georgia, Athens, GA, United States; ^2^ Department of Small Animal Medicine and Surgery, University of Georgia, Athens, GA, United States; ^3^ Sydney School of Veterinary Science, University of Sydney, Camperdown, NSW, Australia; ^4^ K.L. Maddy Equine Analytical Pharmacology Laboratory, School of Veterinary Medicine, University of California Davis, Davis, CA, United States; ^5^ Department of Molecular Biosciences, School of Veterinary Medicine, University of California Davis, Davis, CA, United States

**Keywords:** horse, fentanyl, pharmacokinetics, pharmacodynamics, transdermal, opioid, pain

## Abstract

**Introduction:**

Understanding the pharmacokinetics and pharmacodynamics of fentanyl in horses is crucial for optimizing pain management strategies in veterinary medicine.

**Methods:**

Six adult horses were enrolled in a randomized crossover design. Treatments included: placebo, two 100 mcg/h patches (LDF), four 100 mcg/h patches (MDF), and six 100 mcg/h patches (HDF). Patches were in place for 72 h. Blood was obtained for fentanyl plasma concentration determination, thermal threshold, mechanical threshold, heart rate, respiratory rate, and rectal temperature were obtained prior patch placement and at multiple time points following patch placement for the following 96 h. Fentanyl plasma concentration was determined using LC-MS/MS. Data were analyzed using a generalized mixed effects model.

**Results:**

Mean (range) maximum plasma concentration (Cmax), time to Cmax, and area under the curve extrapolated to infinity were 1.39 (0.82–1.82), 2.64 (1.21–4.42), 4.11 (2.78–7.12) ng/ml, 12.7 (8.0–16.0), 12.7 (8.0–16.0), 12 (8.0–16.0) h, 42.37 (27.59–55.56), 77.24 (45.62–115.06), 120.34 (100.66–150.55) h ng/ml for LDF, MDF, and HDF, respectively. There was no significant effect of treatment or time on thermal threshold, mechanical threshold, respiratory rate, or temperature (*p* > 0.063). There was no significant effect of treatment on heart rate (*p* = 0.364). There was a significant effect of time (*p* = 0.003) on heart rate with overall heart rates being less than baseline at 64 h.

**Conclusions:**

Fentanyl administered via transdermal patch is well absorbed and well tolerated but does not result in an anti-nociceptive effect as measured by thermal and mechanical threshold at the doses studied.

## 1 Introduction

Pain management in horses is currently largely limited to the use of opioids and non-steroidal anti-inflammatory agents (NSAIDs). Indeed, opioids are a mainstay in the management of acute pain in horses (1–3). Unfortunately, most opioids are available only as injectable formulations that require intravenous or intramuscular injections with the need for frequent administration. This treatment approach creates peaks in plasma concentration that may result in adverse effects and nadirs in plasma concentration during which horses may experience pain.

Fentanyl, a pure mu agonist opioid, is available for use in injectable and transdermal patch formulations (4–11). Fentanyl is a highly lipophilic opioid with a rapid onset and brief duration of action (12, 13). In horses, a single intravenous bolus provides an anti-nociceptive effect lasting only 10–30 min. For this reason, the injectable formulation of fentanyl must be administered as a continuous intravenous infusion to maintain the plasma concentrations needed for long-term analgesia (5).

There are two studies in the literature finding an anti-nociceptive effect of fentanyl whose findings differ considerably with respect to the plasma concentration required to achieve this effect. One of these studies (5) was performed using an intravenous bolus dose and an antinociceptive effect was measured via thermal and mechanical threshold in non-painful horses. The other was a clinical study in which fentanyl patches were applied to horses with pain refractory to NSAID therapy (11). In the first study, plasma concentrations of fentanyl measured when an anti-nociceptive effect occurred shortly after the bolus dose (6.1–6.8 ng/ml) (5) were much higher than those achieved with the fentanyl patch in the second study (1.1 ng/ml) (11). This suggests that analgesia may be achieved at lower plasma concentrations when administered transdermally than are required with intravenous administration. Furthermore, fentanyl patches have provided analgesia at concentrations below 1.1 ng/ml in people (0.63 ng/ml) (14) and dogs (0.6 ng/ml) (15), further supporting additional study of the fentanyl patch in horses. Thus, transdermal fentanyl administration represents a potential means of providing continuous opioid mediated analgesia without the need for maintenance of an intravenous catheter or a frequent dosing schedule.

The aims of the study presented here were twofold: (1) to describe the pharmacokinetics of fentanyl administered via transdermal matrix patch at three different doses, and (2) describe the associated pharmacodynamics with regard to anti-nociceptive effect as measured by thermal and mechanical threshold and effect on physical exam parameters. The authors hypothesized that fentanyl would be well absorbed, exhibiting dose dependent pharmacokinetics, and provide a dose dependent anti-nociceptive effect with minimal adverse effects on physical exam variables.

## 2 Materials and methods

### 2.1 Study design

This study was a prospective, randomized, masked, cross-over design. This study was approved by the University of Georgia Institutional Animal Care and Use Committee (Animal Use Protocol A2020 05-010).

### 2.2 Methods

Six horses weighing 528 ± 49 kg (4 males, 2 females) with a mean age of 14 (range 7–23) years were enrolled. Normal health status was confirmed based on physical exam prior to enrollment.

Based on previous pharmacokinetic studies published by the investigators and others in veterinary medicine, data from 6 animals is sufficient to evaluate the interindividual variability of the pharmacokinetic parameters. A sample size calculation with a one-tailed paired t-test found that, on each treatment, four horses would provide sufficient power to detect an effect of 3°C in thermal threshold with a standard deviation of 1.4°C compared to baseline, power of 0.9 and alpha of 0.05 (G*Power 3.1.9.7, Heinrich-Heine-Universität, Düsseldorf, Germany). The additional two horses allowed for some variation in observed values from the hypothetical values used for sample size calculation.

Horses were housed in 12′ × 12′ (3.7 m × 3.7 m) stalls in a temperature-controlled facility for the duration of the study and were acclimatized to the stalls for at least 12 h prior to treatment. Each horse received 1 lb (0.45 kg) of feed and 2–3 flakes of Timothy hay twice daily throughout the study period. All procedures were designed to minimize stress and discomfort to the horses, with continuous monitoring for any signs of adverse effects.

The study was completed in two phases. A fentanyl patch phase for collection of pharmacodynamic and pharmacokinetic data and an intravenous bolus phase to aid in determination of bioavailability of fentanyl from the patch.

#### 2.2.1 Phase I

In the first phase, horses received each of four treatments in a randomly assigned (www.randomizer.org) order with a minimum of a 7-day washout between treatments. These treatments included two 100 mcg/h patches (LDF) (Mylan N.V., Canonsburg, PA) for 72 h, four 100 mcg/h patches (MDF) for 72 h, six 100 mcg/h patches (HDF) for 72 h and placebo (no patches placed). All patches were placed on the metatarsi with half of the patches on each leg. Prior to patch placement, the dorsal aspect of the metatarsi was clipped with a #50 clipper blade and dry gauze was used to wipe away gross debris. All patches were covered with opaque elastic tape (Elastikon, Johnson & Johnson, New Brunswick, NJ). For the placebo treatment, the metatarsi were similarly wrapped to facilitate masking. These fentanyl matrix patch dosages were chosen based on the plasma concentrations achieved by the investigators in a previous study (16), the published range of analgesic plasma concentrations reported for people and dogs (∼0.6 ng/ml) (14, 15), and the analgesic concentrations achieved in horses after intravenous bolus administration (6.1–6.8 ng/ml) with the aim of achieving fentanyl plasma concentrations similar to those reported in both studies (5).

##### 2.2.1.1 Blood collection for pharmacokinetic analysis in first phase

Each horse was weighed and a baseline physical exam was performed prior to each treatment. Horses were instrumented with a 14 gauge intravenous catheters (Mila International, Inc., KY, USA) in one jugular vein for blood collection during the first 24 h of treatment. Subsequent samples were collected via direct venipuncture. Blood samples were obtained at baseline and again at 2, 4, 8, 12, 16, 24, 32, 40, 48, 56, 64, 72, and 96 h after placement. Following removal of a 10 ml waste sample, a total of 6 ml of whole blood was obtained at each timepoint, and stored in lithium heparin tubes (Becton, Dickinson, and Company, NJ, USA) for no longer than 1 h prior to processing. Blood was centrifuged at 1,300 g for 10 min and plasma was harvested and placed in cryovials (VWR, International, PA, USA) prior to storage at −80°C until analysis. All fentanyl patches were labeled and stored at −80°C until analysis to determine the amount of residual fentanyl in the patches.

##### 2.2.1.2 Pharmacodynamic data collection in first phase

Pharmacodynamic data (thermal/mechanical threshold, heart rate, respiratory rate, body temperature, and borborygmi score) were recorded at baseline and the same time points following treatment outlined above for blood sampling. The anti-nociceptive effect of treatment was determined using thermal and mechanical threshold testing (Topcat Metrology Ltd, United Kingdom) over the metacarpus. A coin toss was used to randomize the assigned leg for each unit at each time point. The dorsal aspect of both metacarpi were clipped using a #50 clipper blade to ensure good contact. For the thermal threshold device, a heating element was applied over the shaved area and connected to a control unit secured to the horse's withers via a surcingle. A masked operator using a wireless remote increased the temperature by 0.8°C/s until the horse exhibited an avoidance behavior (stomping, kicking, sniffing of the stimulated forelimb). The temperature at which this avoidance behavior occurred was the thermal threshold for that time point. A maximum temperature of 55°C was not exceeded in order to avoid tissue injury. For the mechanical threshold device, an actuator with a 1 × 1 mm pin was attached to the shaved metacarpus opposite that of the thermal threshold device. This device was controlled by a masked operator increasing the pressure exerted by the pin until an avoidance behavior was observed (stomping, kicking, sniffing of the stimulated limb). The pressure in Newtons (N) at which this behavior occurred was the mechanical threshold for that time point. A maximum pressure of 20 N was not exceeded in order to avoid tissue injury. Baseline thermal and mechanical thresholds were obtained in triplicate prior to treatment administration. Single measurements were obtained for each subsequent measurement following treatment. Physical exam variables were recorded at each timepoint prior to measurement of thermal and mechanical threshold. Borborygmi was scored using a previously published scoring system (17) assigning a score to each quadrant following 1 min of auscultation with the sum of these values being the total score for that timepoint.

#### 2.2.2 Phase II

At least 30 days following the first phase, all horses underwent the second phase of treatment which included a single 2 mg fentanyl bolus administered intravenously to aid in determination of fentanyl bioavailability from the patch. For this treatment, horses were instrumented with 14-gauge intravenous catheters in both jugular veins prior to treatment. One catheter was used for administration of treatment and immediately removed following treatment. The catheter in the opposite jugular vein was used for sampling. Following collection of a 10 ml waste sample, a total of 6 ml of whole blood was obtained prior to treatment and again at 1, 3, 5, 7, 10, 20, 30, 40, 50, 60, 90, 120, 150, 180, 240, 300, 360, and 420 min following fentanyl administration. The sampling catheter was removed following collection of the final sample.

### 2.3 Fentanyl concentration determination

Plasma calibrators were prepared by dilution of the fentanyl working standard solutions (Cerilliant, Round Rock, TX) with drug free equine plasma to concentrations from 0.005 to 50 ng/ml. Calibration curves and negative control samples were prepared fresh for each quantitative assay. Quality control samples were included with each sample set as an additional check of accuracy.

Prior to analysis, 400 µl of plasma samples were diluted with 100 µl of water containing 4 ng/ml of d5-fentanyl internal standard (Cerilliant, Round Rock, TX), and 2 ml 0.1M phosphate buffer at pH 7 and vortexed briefly to mix. The samples were subjected to solid phase extraction using Cerex PolyChrom Clin II (35 mg/ 3cc) columns (Tecan SPE Inc., Baldwin Park, CA). Plasma samples were loaded on and passed through the columns using a CEREX system 48 Processor with positive pressure SPE manifold (SPE Ware, Baldwin Park, CA). A minimum of 2 min was allowed for samples to pass through the column. Subsequently, the columns were rinsed consecutively with 3 ml of water, 2 ml 1M acetic acid, and 3 ml methanol prior to elution with 2 ml of methanol:ammonium hydroxide (97:3, v:v). Samples were dried under nitrogen, reconstituted in 120 µl of redissolve solution, 10% ACN in water with 0.2% formic acid and 30 µl injected into the liquid chromatography tandem mass spectrometry (LC-MS/MS) system.

Quantitative analysis was performed on a TSQ Altis triple quadrupole mass spectrometer coupled with a Vanquish liquid chromatography system (Thermo Scientific, San Jose, CA). The spray voltage was 3,500 V, the vaporizer temperature was 350°C, and the sheath and auxiliary gas were 50 and 10 respectively (arbitrary units). The standards were infused into the instrument to optimize product masses and collision energies of the analytes. Chromatography employed an ACE 3 C18 5 cm × 2.1 mm column (Mac-Mod Analytical, Chadds Ford, PA) and a linear gradient of acetonitrile (ACN) in water containing 0.2% formic acid, at a flow rate of 0.35 ml/min. The initial ACN concentration was held at 5% for 0.2 min, ramped to 95% over 3.8 min and held at that concentration for 0.2 min, before re-equilibrating for 2.9 min at initial conditions.

Detection and quantification was conducted using selective reaction monitoring (SRM) of initial precursor ion for fentanyl [mass to charge ratio *(m/z)* 337.2] and the internal standard d5-fentanyl (*(m/z)* 342.2). The response for the product ions for fentanyl (*m/z* 105.2, 132.2, 188.2) and the internal standard d5-fentanyl (*m/z* 102.9, 104.9, 188.0) were plotted and peaks at the proper retention time integrated using Quanbrowser software (Thermo Scientific). Quanbrowser software was used to generate calibration curves and quantitate fentanyl in all samples by linear regression analysis. A weighting factor of 1/X was used for all calibration curves.

Patch Analysis: Fentanyl patches were cut into 1 cm portions and mixed in 100 ml of methanol and subsequently serially diluted tenfold in redissolve solution three times. The samples were quantitated with a calibration curve prepared in the redissolve solution. Ten microliters of the sample was injected in the LC-MS/MS system utilizing the analytical conditions described for plasma samples. Total dose absorbed from the patch was determined by subtracting the residual amount of fentanyl in the patch from the total fentanyl in the patch formulation (10 mg per patch).

### 2.4 Pharmacokinetic analysis

The peak concentration (C_max_) and time to peak plasma concentration (T_max_) were determined by visual inspection of the concentration-time data. For determination of initial estimates for subsequent model fitting, non-compartmental analysis (NCA), using a commercially available computer software program (Phoenix Winnonlin v8.3, Certara, Princeton, NJ) was used. Subsequent to NCA, a nonlinear mixed effects modeling (NLME) approach with the Phoenix NLME software program was used to fit a compartmental model to the data. The first-order conditional estimation method with interaction (FOCE-ELS) was used in the model-building process. Both two and three compartment models were evaluated. For residual error models, additive, multiplicative, and Poisson error models were all considered. Random effects were included for all structural variables and were modeled with log linear functions. A diagonal variance-covariance matrix was used for the random effects. In assessing which model provided the best fit, visual analysis of the observed vs. predicted concentration graphs, residual plots, Akaike Information Criterion, %CV, and -2LL were considered. A simultaneous fit of the intravenous and transdermal data was attempted but the fit was poor. Transdermal data were too variable and had inadequate frequency of sampling to fully determine the elimination phase using a population PK approach. For transdermal administration, pharmacokinetic parameters from NCA are reported.

Bioavailability was calculated for individual horses using the formula:

$$\left(\mathrm{AU}C_{\mathrm{transdermal}}/\mathrm{Dos}e_{\mathrm{transdermal}}\right)/\left(\mathrm{AU}C_{\mathrm{IV}}/\mathrm{Dos}e_{\mathrm{IV}}\right)$$

where the transdermal dose is the total amount absorbed determined by analysis of the patches. The bioavailability was calculated for each horse within a dose group and the individual values averaged and reported.

### 2.5 Statistical analysis

All analyses were performed using JMP Statistical Discovery (Cary, NC). Normality of the data was assessed by examination of histograms and normal Q-Q plots of residuals and Shapiro Wilk tests. Pharmacokinetic parameters were analyzed with generalized linear mixed model with treatment as a fixed effect and horse as a random effect. Where a significant effect treatment was found, a Tukey test was used for multiple comparisons between treatments. Heart rate, respiratory rate, temperature, and borborygmi scores were analyzed using a generalized linear mixed effects model with treatment, time, and the interaction of treatment and time as fixed effects. Horse was included as a random effect. A Dunnett's test for multiple comparisons to baseline was utilized for variables in which the mixed effects model found a significant effect of time. Generalized linear mixed models were selected due to their ability to handle the correlated data structure inherent in crossover study designs and to account for inter-individual variability. Figures were generated with GraphPad Prism. For all analyses, *p* < 0.05 was considered statistically significant.

## 3 Results

### 3.1 Fentanyl concentration determination

The response was linear and gave correlation coefficients of 0.99 or better. Accuracy was reported as percent nominal concentration and precision as percent relative standard deviation. Accuracy was 97% for 0.0075 ng/ml, 112% for 2 ng/ml and 102% for 25 ng/ml. Precision was 12% for 0.0075 ng/ml, 3% for 2 ng/ml and 3% for 25 ng/ml. The technique was optimized to provide a limit of quantitation (LOQ) of 0.005 ng/ml and a limit of detection (LOD) of approximately 0.0025 ng/ml in plasma and an LOQ of 0.01 ng/ul and an LOD of approximately 0.005 ng/ul for the patches.

### 3.2 Pharmacokinetics

**Figure 1:**
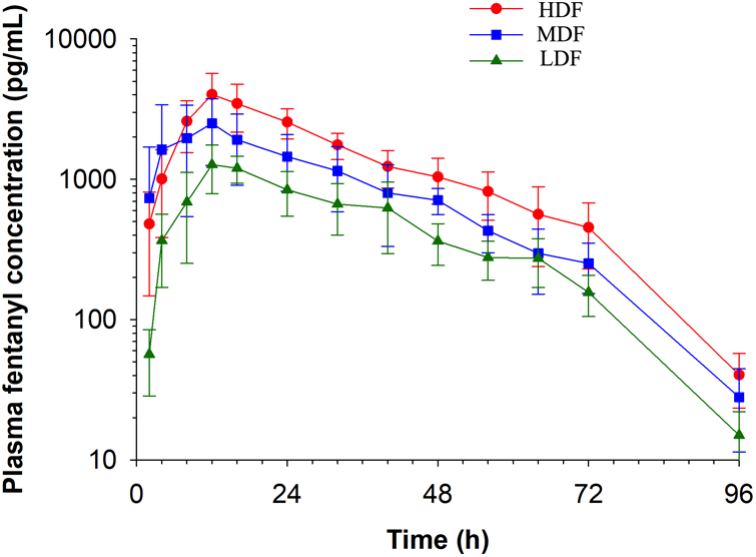
Fentanyl plasma concentration (mean ± SD) over time following application of two (LDF), four (MDF) and six (HDF) 100 mcg/h (16.8 mg/patch) matrix patches to healthy adult horses (*n* = 6).

Plasma concentrations of fentanyl for HDF, MDF, and LDF are depicted in Figure 1. Pharmacokinetic variables following non-compartmental analysis (NCA) for all patch treatments are presented in Table 1. The maximum plasma concentration (Cmax) of HDF was significantly higher than MDF (*p* = 0.048) and LDF (*p* = 0.001). There was no difference in Cmax between MDF and LDF (*p* = 0.089). The area under the curve extrapolated to infinity (AUCinf) of HDF was significantly greater than MDF (*p* = 0.002) and LDF (*p* = <0.001); and MDF was significantly greater than LDF (*p* = 0.008). There was no difference between groups in time to maximum plasma concentration (Tmax) (*p* > 0.89), terminal half-life (HL *λ_Z_*) (*p* > 0.586), clearance (*p* > 0.304) for all, or terminal slope of the plasma concentration time curve (*λ_Z_*) (*p* > 0.686).

**Table 1:** Pharmacokinetic parameters (mean and range) generated by non-compartmental analysis for fentanyl following placement of two (LDF), four (MDF), and six (HDF) 100 mcg/h matrix patches in healthy adult horses (*n* = 6).

	LDF	MDF	HDF
C_max_ (ng/ml)	1.39 (0.82−1.82)	2.64 (1.21–4.42)	4.11 (2.78−7.12)^a^
T_max_ (h)	12.7 (8.0–16.0)	12.7 (8.0–16.0)	12 (8.0–16.0)
AUC_inf_ (h ng/ml)	42.37 (27.59–55.56)	77.24 (45.62–115.06)^a^	120.34 (100.66–150.55)^a^^,^^b^
AUC % extrap	0.435 (0.170–0.821)	0.805 (0.088–2.27)	0.448 (0.200–0.846)
HL *λ*_Z_ (h)	7.94 (6.37–11.4)	9.73 (6.50–15.9)	9.18 (6.71–12.2)
λ_Z_ (1/h)	0.091 (0.061–0.109)	0.080 (0.044–0.107)	0.079 (0.057–0.103)
Cl/F (ml/min/kg)	15.57 (11.36–22.88)	9,929.3 (5,794.0–14,608.8)	16.01 (12.58–18.81)
Vd/F (L)	5,558.9 (3,535.0–7,202.2)	9,098.9 (3,260.4–16,768.9)	6,583.3 (4,706.2–7,694.1)

C_max_, maximum plasma drug concentration; T_max_, time of maximum plasma drug concentration; AUC_inf_, area under the curve extrapolated to infinity; AUC % extrap, percent of area under the curve extrapolated; HL λ_Z_, terminal half-life; λ_Z_, terminal slope of the plasma concentration time curve; Cl/F, systemic clearance per fraction absorbed for patch treatments, total systemic clearance for IV treatment; Vd/F, volume of distribution per fraction absorbed for patch treatments and volume of distribution at steady state for intravenous treatment.

^a^ Significantly greater than LDF (*p* < 0.008, for both).

^b^ Significantly greater than MDF (*p* = 0.002).

A 3-compartment model and a multiplicative residual error gave the best fit to the plasma concentration data from the intravenous administration. The diagnostic plots, used to assess the fit for the NLME model are provided as Supplementary Figures 1, 2. The pharmacokinetic parameters (estimate and % coefficient of variation for the fixed and random effects) for the NLME model are shown in Table 2.

**Table 2:** Model typical values (tv) for fentanyl following a single intravenous (2 mg) administration to horses (*n* = 6).

Parameter	Estimate	CV (%)
tvA (pg/ml)	12,661.7	19.4
tvB (pg/ml)	6,613.1	23.0
tvC (pg/ml)	528.6	27.1
tvAlpha (1/h)	17.8	34.8
tvBeta (1/h)	2.12	15.4
tvGamma (1/h)	0.375	12.5
t_1/2*α*_ (h)	0.039	34.8
t_1/2*β*_ (h)	0.327	15.4
t_1/2*γ*_ (h)	1.85	12.5
AUC_last_ (h*pg/ml)	5,243.4	5.39
Cl (ml/h/kg)	722.4	5.39
V1 (L/kg)	0.191	17.1
V2 (L/kg)	0.185	10.1
V3 (L/kg)	0.351	21.3
stdev0	0.150	10.9
Between subject variability (%CV)		
A	0.104	33.1
B	0.059	24.5
C	0.209	48.2
Alpha	1.48 × 10−6	0.12
Beta	0.004	5.92
Gamma	0.035	18.9

tvA, tvB and tvC, intercepts at *t* = 0 for the model equation; tvalpha, tvbeta and tvgamma, slopes for the modeled equation; *V*_1_, *V*_2_, *V*_3_, volumes of the central, second and third compartments, respectively; Vss, volume of distribution at steady state (calculated as MRT * Cl); t1_/2α_, phase 1 half-life; t_1/2β_, phase 2 half-life; t_1/2γ_, phase 3 half-life; AUC_last_, area under the curve until the last time point; Cl, total serum clearance. stdev0 = the estimated residual standard deviation for plasma data.

**Figure 2:**
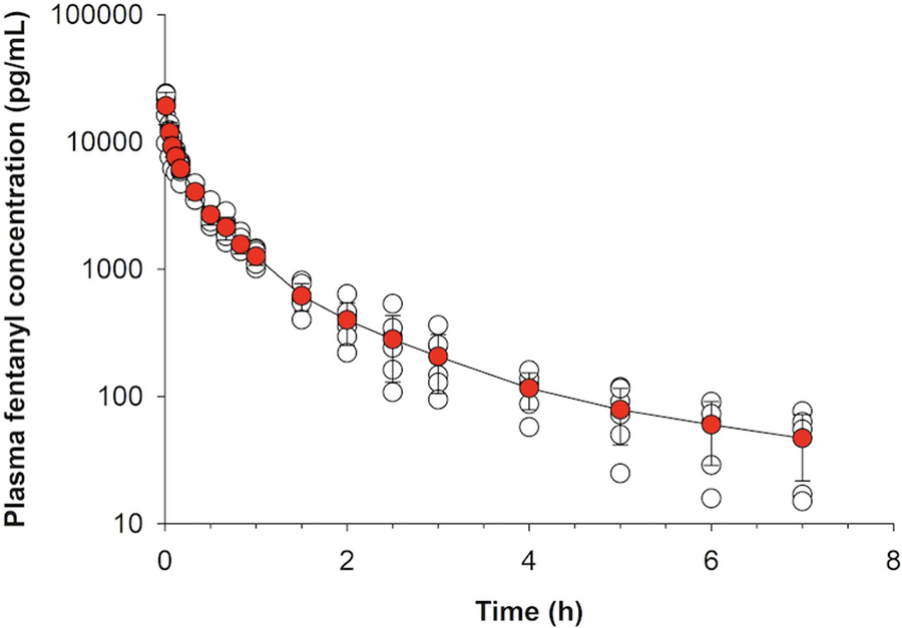
Fentanyl plasma concentration (mean ± SD) over time following intravenous administration of 2 mg fentanyl to healthy adult horses (*n* = 6).

Plasma concentrations from the intravenous treatment group are presented in Figure 2. Total dose absorbed from the patch (Dose_patch_) and calculated bioavailability are presented in Table 3. There was no significant difference between groups in bioavailability of the fraction absorbed from the patch (*p* > 0.346 for all).

**Table 3:** Mean ± SD dose of fentanyl absorbed and associated bioavailability after application of two (LDF), four (MDF) and six (HDF) 100 mcg/h (16.8 mg/patch) matrix patches to healthy adult horses (*n* = 6).

Treatment	Target dose (mg)	Dose absorbed (mg)	Fractional bioavailability (%)
LDF	33.6	28.8 ± 4.8	57.8 ± 5.7
MDF	67.2	57.9 ± 7.1	49.9 ± 14.0
HDF	100.8	86.0 ± 6.6	54.3 ± 8.6

### 3.3 Pharmacodynamics

All treatments were well tolerated. There were no adverse effects of treatment noted and no horse showed signs of colic at any time. All patches remained well adhered to the skin until the time of patch of removal. There was no evidence of irritation of the skin at the treatment site following patch removal.

#### 3.3.1 Thermal and mechanical threshold

**Figure 3:**
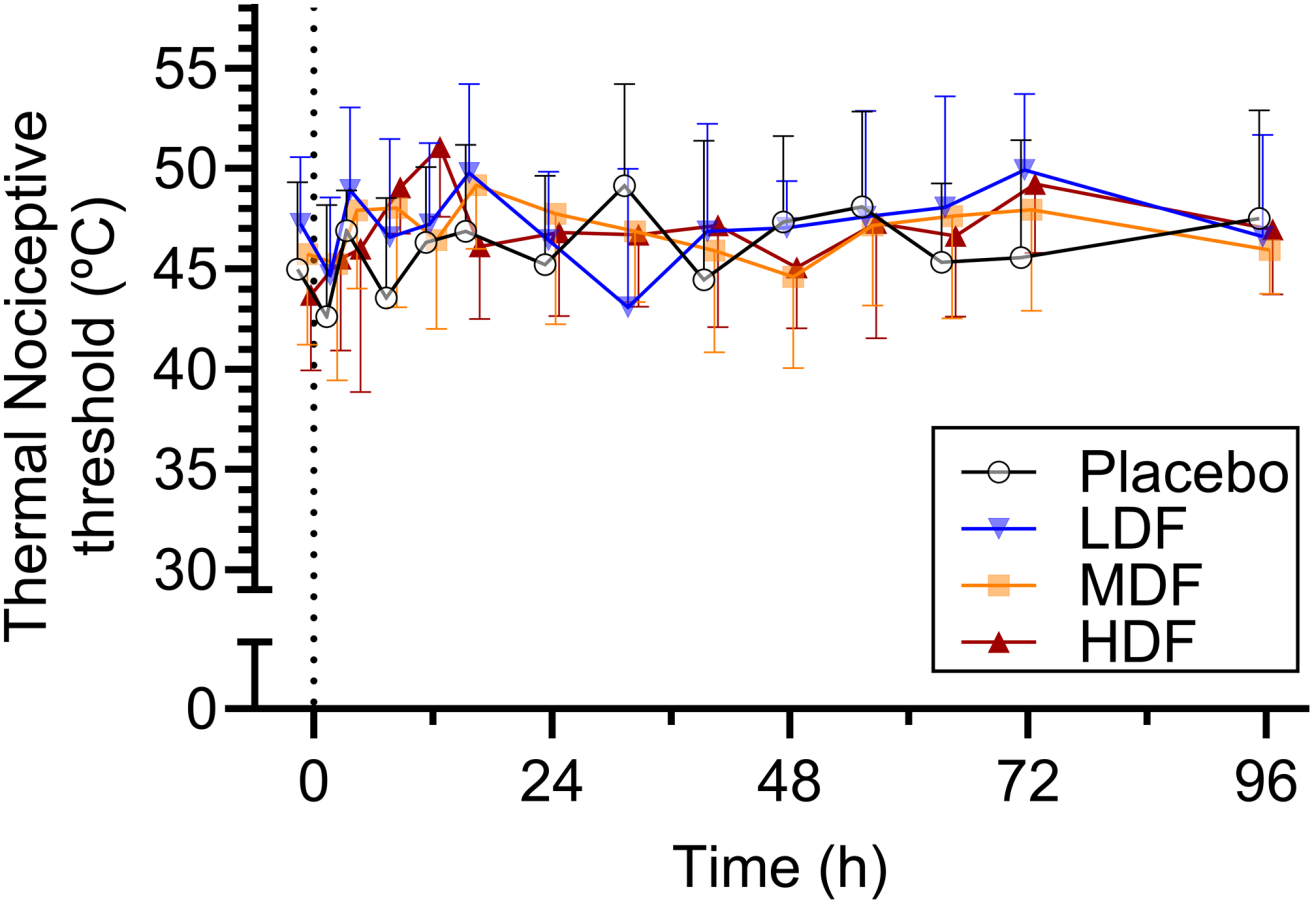
Thermal nociceptive thresholds (°C) (mean ± SD) at from 0 to 96 h following application of zero (placebo), two (LDF), four (MDF) and six (HDF) 100 mcg/h (16.8 mg/patch) matrix patches to healthy adult horses (*n* = 6).

**Figure 4:**
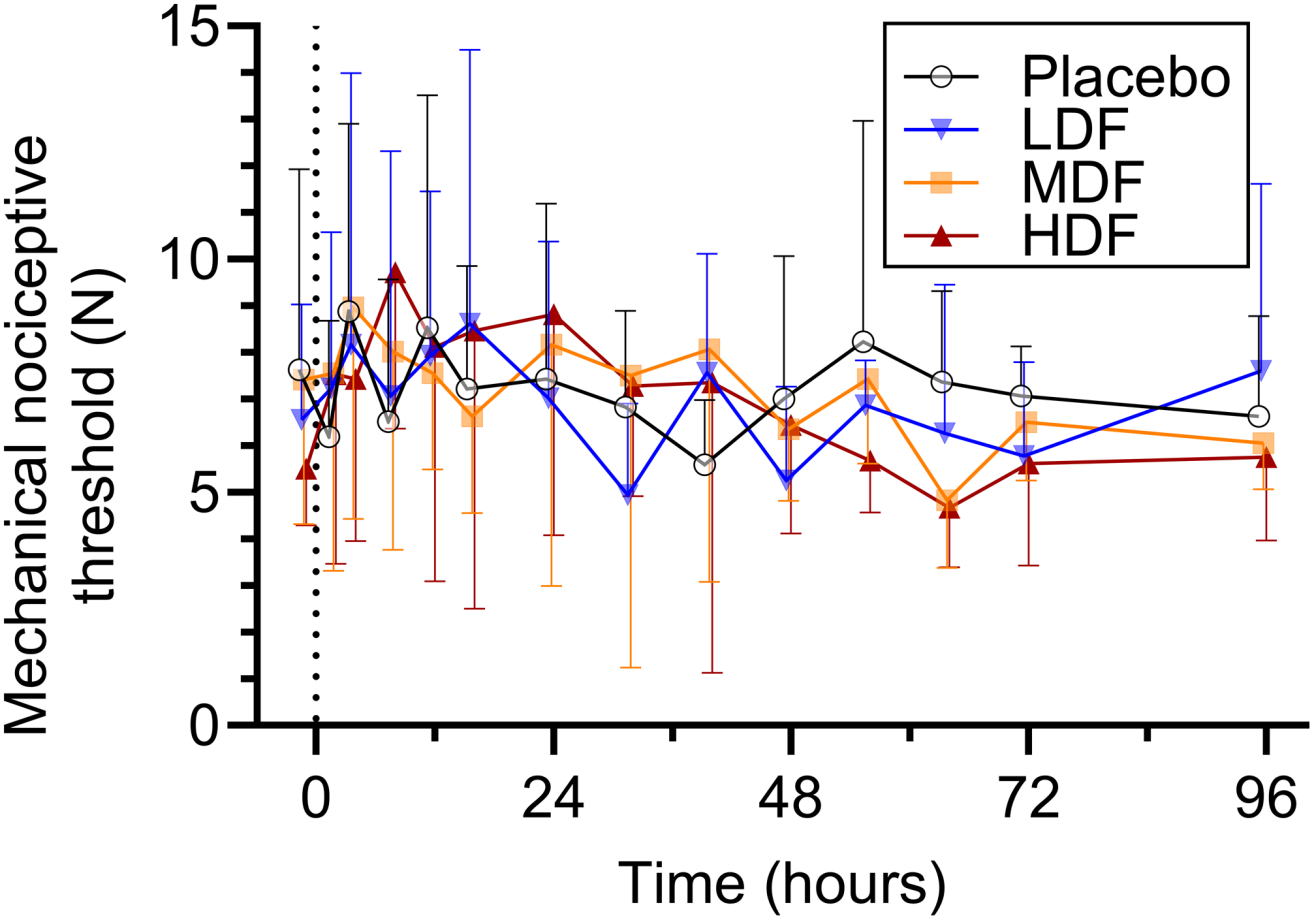
Mechanical nociceptive thresholds (°C) (mean ± SD) at from 0 to 96 h following application of zero (placebo), two (LDF), four (MDF) and six (HDF) 100 mcg/h (16.8 mg/patch) matrix patches to healthy adult horses (*n* = 6).

Ambient temperature during the study period ranged between 19.9°C and 22.1°C (mean ± standard deviation, 20.6 ± 0.4°C). Mean thermal and mechanical threshold over time are presented in Figures 3, 4, respectively. For thermal threshold, there was no significant effect of treatment (*p* = 0.418), time (*p* = 0.063), or their interaction (*p* = 0.457). For mechanical threshold, there was a significant effect of time (*p* < 0.001). However, a *post-hoc* Dunnett's test for multiple comparisons revealed no significant difference from baseline at any timepoint. There was no effect of treatment (*p* = 0.437) or the interaction of treatment and time (*p* = 0.698).

#### 3.3.2 Physical exam variables

In regard to heart rate, least squares mean (LSM) and 95% confidence interval [CI (LL, UL)] were 40 [30, 43], 37 [34, 41], 35 [32, 39], and 38 [34, 42] beats per minute for LDF, MDF, HDF, and P, respectively. There was a significant effect of time (*p* = 0.003). *Post hoc* Dunnett's test for multiple comparisons revealed heart was significantly lower than baseline at 64 h (*p* = 0.006) only. There was no significant effect of treatment (*p* = 0.3644) or the interaction of treatment and time (*p* = 0.579).

LSM and 95% CI respiratory rate was 14 [12, 17], 12, [9, 14], 11 [8, 13], and 13 [11, 15] breaths per minute for LDF, MDF, HDF, and P, respectively. There was a significant effect of time (*p* < 0.001). However, *post-hoc* Dunnett's test for multiple comparisons revealed no significant difference from baseline at any timepoint. There was no significant effect of treatment (*p* = 0.072) or the interaction of treatment and time (*p* = 0.385).

LSM and 95% CI rectal temperature was 99.0 [98.5, 99.6], 99.3 [98.7, 99.8], 99.5 [98.9, 100.0], and 99.4 [98.8, 99.9]°F for LDF, MDF, HDF, and P, respectively. There was a significant effect of time (*p* < 0.001). *Post hoc* Dunnett's test revealed no significant difference from baseline at any timepoint. There was no effect of treatment (*p* = 0.560) or the interaction of treatment and time (*p* = 0.532).

LSM and 95% CI borborygmi scores were 13 [12, 14], 12 [12, 13], 12 [12, 13], and 13 [12, 14] for LDF, MDF, HDF, and P, respectively. There was no effect of treatment (*p* = 0.155), time (*p* = 0.230), or the interaction of treatment and time (*p* = 0.744).

## 4 Discussion

### 4.1 Pharmacokinetics

In the current study, we chose to model the intravenous data using a population pharmacokinetic model, which differs from previous reports of fentanyl pharmacokinetics in the horse. This modeling approach allowed for reporting of inter-individual variability for the pharmacokinetic parameters as well providing an overall estimate of residual variability. While the model fit in the current study was good, it is important to note that that the number of horses included in the current study is low and the model could be strengthened by the addition of more animals. Although modeling of transdermal data with a population model was attempted, the fit was poor and therefore, NCA was used for this data set.

In the present study, fentanyl administered via a transdermal matrix patch exhibited a dose dependent Cmax and AUCinf with similar terminal half-lives and clearance rates across all treatments. The pharmacokinetics of a single dose of fentanyl administered via a matrix patch have been previously described in a study investigating the effect of patch location by Skrzypczak et al. (16). In that study, fentanyl from two 100 mcg/h patches was well absorbed at the metacarpus, tail, and inguinal abdominal region. The Cmax reported in that study ranged from 1.55 to 2.07 ng/ml which is higher than the 1.378 ng/ml achieved with placement of two 100 mcg/h patches in the present study. Indeed, the decision to apply two, four, and six 100 mcg/h patches was based on the results of this previous study with the hope that six patches would result in plasma concentrations in excess of the 6.1–6.8 ng/ml reported to provide an antinociceptive effect (5). The lower plasma concentrations reported here could be due to placement of the patch on the metatarsus, as opposed to one of the previously studied locations, suggesting there may be site-dependent absorption variability associated with the metatarsus in comparison to the previously studied sites. However, there is not enough room to place six patches on the ventral tail base, ensuring continuous good contact of six patches in the inguinal region for 72 h would have been difficult to achieve while maintaining masking of observers, and the metacarpus was occupied by the thermal and mechanical threshold units. Therefore, these locations were not chosen for patch placement. The Tmax reported here was 12 h for all groups, which is consistent with the previous study reporting a Tmax of 10–14 h.

The AUCinf of 42.374 ng h/ml was slightly lower in the present study than the previous study that described 44.6–46.6 ng h/ml with placement of two patches (16). This is most likely attributed to differences in absorbance of fentanyl from the patch at the metatarsal location. The previous study did not determine how much fentanyl remained in the patch following removal, and therefore it is impossible to say if the horses received the same fraction of the dose incorporated into the patch.

Comparing matrix patches to reservoir patches, it appears that reservoir patches may be superior in regard to exposure to fentanyl. Indeed, placement of two 100 mcg/h reservoir patches resulted in a Cmax of 2.6 ng/ml and AUCinf of 80–92 ng h/ml (8). However, differences in study design could account for this difference. This superior absorption from the reservoir patches may be attributed to the fact that the skin was clipped and shaved prior to patch application which could have resulted in damage or removal of the stratum corneum, the rate limiting barrier in the absorption of transdermal fentanyl (18). Additionally, reservoir patches that became dislodged before 24 h of application were replaced with a new patch which may have contributed to the higher Cmax and AUCinf in that study compared to that reported for fentanyl matrix patches.

Compared to intravenous administration, the terminal half-life following transdermal administration is prolonged. Although this interpretation should be made with caution, as the minimum number of data points (3–4) were used in the calculation of the terminal slope, this suggests that flip-flop kinetics occurs with transdermal administration of fentanyl matrix patches in horses.

### 4.2 Pharmacodynamics

Previous studies examining the effect of fentanyl on thermal threshold in horses have yielded conflicting results. Intravenous bolus administration of a 2.5, 5, and 10 mcg/kg fentanyl resulted in a dose dependent increase in thermal threshold using a radiant thermal stimuli, although fentanyl plasma concentrations were not reported in that study (6). A later study, using the same intravenous fentanyl dosages as the previous study described an increase in thermal threshold measured at the withers following administration of 10 mcg/kg, corresponding to a plasma concentration of 6.1–6.8 ng/ml (5). Conversely, stepped infusions of fentanyl did not result in a significant difference in thermal threshold from placebo at plasma concentrations as high as 7.82 ng/ml (9). These conflicting results may be due to differences in study design. In the latter study, the thermal threshold stimulus had an automatic cutout at 45°C as opposed to 56°C in the former study utilizing the same thermal stimulus model. The lower cutout temperature may have resulted in the inability of the investigators to accurately report the thermal threshold of the horses with higher fentanyl plasma concentrations due to blunting of the data by the low cut out temperature. In the study reported here, an automatic cutout of 55°C was applied and therefore any effect of fentanyl on thermal threshold should have been captured. Moreover, the lack of effect on thermal threshold observed here is likely due to the lower-than-expected plasma concentration achieved in the HDF group (4.11 ng/ml).

Only a single other study has examined the effect of fentanyl on mechanical threshold in horses. In that study, there was a significant increase in mechanical threshold 10 min following administration of 10 mcg/kg. However, the effect was not observed at 30 min following administration (5). No effect of fentanyl on mechanical threshold was found in the present study and this is likely due to the lower fentanyl plasma concentration. The absence of a significant anti-nociceptive effect, despite well-tolerated treatments, underscores the need for further investigation into the dose-response relationship of transdermal fentanyl in horses.

Fentanyl was generally well tolerated in all horses in the present study. There was no effect of fentanyl treatment on any physiologic variable studied, with the exception of a decreased heart rate overall at 64 h following treatment. This time point was at midnight so this effect may simply be due to a circadian effect. Previous studies of fentanyl in horses administered either intravenously or via transdermal patch have either found no difference in physiologic variables (5, 8, 16) or increases in heart rate, respiratory rate (9), and rectal temperature (8) only at high plasma concentrations.

Pure mu agonist opioids are known to decrease gastrointestinal motility via activity at opioid receptors in the myenteric plexus of the gastrointestinal tract. An *in vitro* study of the effect of different opioids on motility of equine intestine revealed that this effect is mediated by activity at kappa and not mu receptors (19). However, in that study, fentanyl decreased motility but this effect was not reversed by administration of mu or kappa antagonists, leading the authors to conclude that the effect of fentanyl on gut motility is more likely attributed to the antimuscarinic effects of this drug. Nevertheless, in the present study, all horses continued to defecate, showed no signs of colic, and borborygmi scales were unaffected by treatment. These findings are in agreement with other studies of fentanyl in horses (5, 8, 9, 16).

This study does suffer from limitations. The small sample size may affect the generalizability of the findings reported here. Additionally, as with all studies utilizing a thermal and/or mechanical threshold technique, it is possible that this model does not accurately reflect the type of pain that horses experience clinically associated with surgery and inflammation. Further studies utilizing clinical models of pain are requisite to fully describe the potential of fentanyl as an analgesic in horses. Additionally, the plasma concentrations achieved were far lower than were targeted in the study design. The number of patches applied for each treatment were based on plasma concentrations achieved in previous studies using the same type of fentanyl matrix patch (16) with the aim of achieving the plasma concentration that was previously described as anti-nociceptive (5). If an additional eight or ten patch treatment group were included, then that plasma concentration may have been achieved and an anti-nociceptive effect observed.

## 5 Conclusion

Our study demonstrates that while fentanyl patches are well-tolerated in horses, the doses studied did not achieve an anti-nociceptive effect. These findings highlight the need for further research to identify effective analgesic strategies using transdermal fentanyl. Future studies should explore higher doses of fentanyl, alternative patch locations, or clinical pain models that more closely resemble post-operative pain in horses.

## Data Availability

The raw data supporting the conclusions of this article will be made available by the authors, without undue reservation.
