# Supplementary material for: The pharmacokinetics and pharmacodynamics of fentanyl administered via transdermal patch in horses
Source: Front Pain Res (Lausanne). 2024 Mar 20;5:1373759. doi: 10.3389/fpain.2024.1373759 (PMC10987731; doi:10.3389/fpain.2024.1373759)
Supplement: Supplementary file 1 [file Datasheet1.pdf]

Supplementary Figures:

A.) B.)

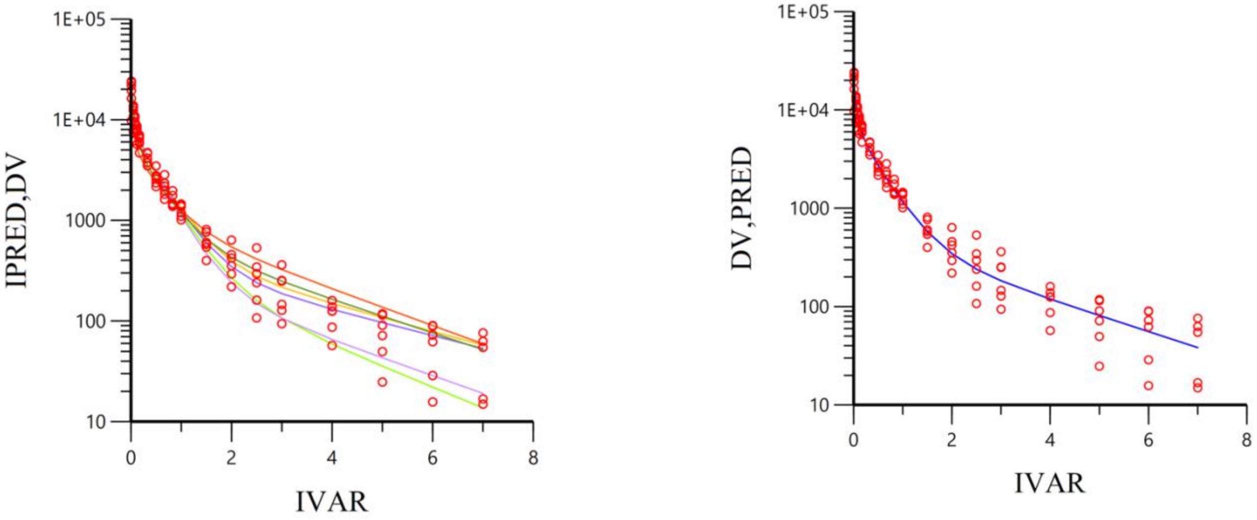

Supplementary Figure 1. (A) Plot of dependent variable (DV; concentrations) and individual prediction (IPRED) vs independent variable (IVAR; time) (B) Plot of dependent variable (DV; concentrations) and population prediction (PRED) vs the independent variable (IVAR: time).

A.) B.)

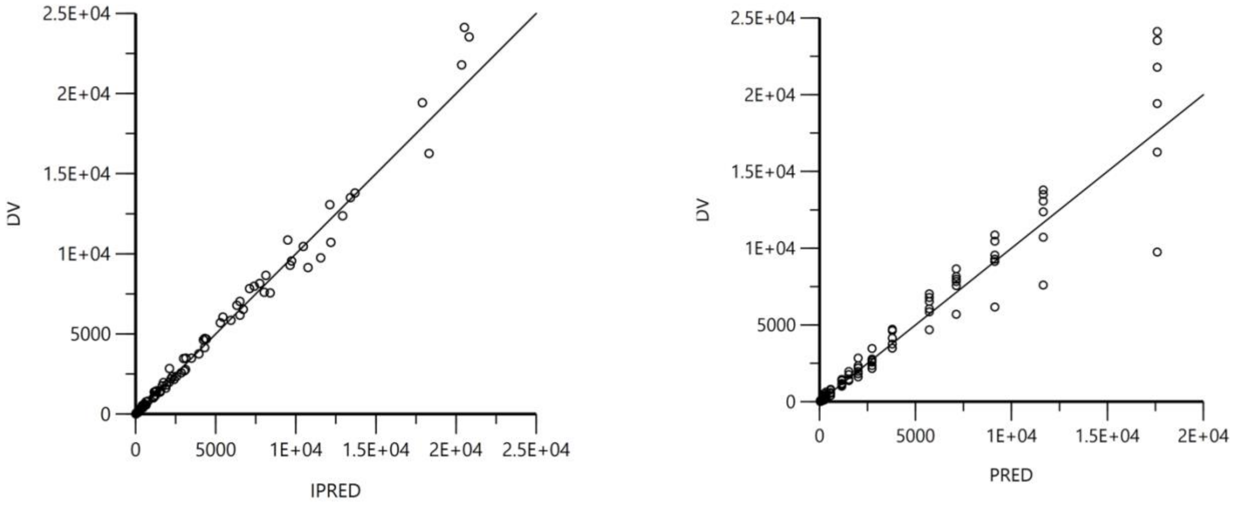

Supplementary Figure 2. (A) Plot of the dependent variable (DV; concentrations) versus individual predicted values (IPRED; predicted concentrations) and (B) Plot of the dependent variable (DV; concentrations) versus population predictions (PRED)
